# Caregiver awareness of pediatric respiratory red-flag signs and emergency activation intentions: a province-wide cross-sectional study in China

**DOI:** 10.3389/fmed.2026.1702865

**Published:** 2026-03-12

**Authors:** Ling Li, Zhengtao Li

**Affiliations:** Pediatric Neurology and Respiratory Medicine, The Central Hospital of Enshi Tujia and MiaoAutonomous Prefecture, Enshi, Hubei, China

**Keywords:** caregiver knowledge, emergency activation intention, emergency medical services, health literacy, pediatric respiratory danger signs

## Abstract

**Background:**

Caregiver recognition of pediatric respiratory red-flag signs remains suboptimal despite established international guidelines. The purpose of this study was to investigate caregiver awareness of pediatric respiratory red-flag signs and their intended emergency activation behaviors in China.

**Methods:**

We conducted a cross-sectional, mixed-mode survey involving 2,702 caregiver–child dyads across pediatric outpatient departments, emergency departments, and wards in Hubei Province, China. Data were collected using a validated questionnaire that measured awareness of 12 red-flag respiratory signs and emergency activation intentions across 5 clinical vignettes. Primary outcomes were adequate awareness (Awareness Index ≥0.70) and high emergency activation intention (Emergency Activation Intention Score (EAIS) ≥ 20). Data were analyzed using multivariable logistic regression.

**Results:**

Adequate awareness was achieved by only 22.9% (*n* = 618) of the caregivers. Notably, symptom recognition varied substantially: identification rates were relatively high for cyanosis (71.0%) and severe chest indrawing (68.4%), but markedly lower for very low oxygen saturation (36.6%) and apnea (40.7%). Conversely, a majority of the cohort (*n* = 1,756, 65.0%) demonstrated appropriate emergency activation intentions. Caregivers exhibited a high propensity to contact emergency services during urgent scenarios (86–89%) while exercising suitable restraint during non-urgent presentations (12.3%). Multivariable analysis identified college education as the most robust predictor of adequate awareness (aOR = 3.41, 95% CI: 2.75–4.23, *p* < 0.001), followed by knowledge of the emergency number 120 (aOR = 1.67, *p* = 0.004) and a history of prior respiratory hospitalization (aOR = 1.34, *p* = 0.015). Furthermore, a distinct dose–response relationship regarding health literacy was observed, with adequate awareness being significantly more prevalent among caregivers with high health literacy compared to those with low health literacy (29.8% vs. 15.1%, *p* < 0.001). Finally, emergency activation rates were influenced by the clinical setting, as caregivers in the emergency department demonstrated significantly higher rates than those in outpatient environments (70.7% vs. 62.8%, *p* < 0.001).

**Conclusion:**

Despite appropriate emergency activation intentions, caregivers’ limited recognition of red-flag symptoms highlights a critical knowledge–behavior gap. Targeted educational interventions addressing these specific deficits are essential to improving pediatric respiratory emergency outcomes.

## 1 Introduction

Acute childhood bronchitis, while usually a self-limited viral infection, is clinically important because some children develop respiratory compromise that necessitates emergency medical services (EMS) (1–3). In line with pediatric cough and respiratory guidelines, we operationally define acute bronchitis in children as an acute lower respiratory tract infection characterized by cough, often with a preceding upper respiratory tract infection, lacking radiographic findings of pneumonia, and typically resolving within a few weeks in otherwise healthy children (4–7). This definition aligns with contemporary pediatric clinical practice guidelines, which distinguish acute bronchitis (affecting children of all ages within our 0–12 year study population) from acute bronchiolitis, defined as inflammation of the small airways (bronchioles) occurring predominantly in infants and young children <24 months of age, characteristically presenting with wheezing, tachypnea, and respiratory distress due to small airway obstruction (6, 8, 9). While acute bronchitis and asthma exacerbations (characterized by recurrent, reversible airflow obstruction and bronchodilator responsiveness) are distinct entities, the frequent symptom overlap—cough, wheeze, and dynamic respiratory trajectories—among these conditions complicates timely caregiver recognition of deterioration (10, 11). Consistent with pediatric practice, we therefore use the term “acute bronchitis” cautiously and pragmatically to describe a cough-predominant illness without radiographic pneumonia, acknowledging that many young children with viral lower respiratory infections may instead satisfy criteria for bronchiolitis or early asthma. Critically, regardless of the specific underlying diagnosis, whether uncomplicated bronchitis, evolving bronchiolitis in an infant, or an asthma exacerbation, the emergence of red-flag respiratory signs (e.g., severe chest indrawing, cyanosis, apnea, and stridor at rest) mandates urgent caregiver recognition and emergency care activation (12, 13). In this study, “acute bronchitis” thus serves as a familiar clinical context for our vignettes, while the primary analytic focus remains on caregiver recognition of red-flag respiratory signs across the spectrum of acute pediatric respiratory presentations.

International pediatric guidance codifies red-flag signs that should trigger urgent referral. Within the World Health Organization Integrated Management of Childhood Illness (IMCI) framework, inability to drink, persistent vomiting, convulsions, lethargy or unconsciousness, stridor when calm, and lower chest indrawing are key indicators of severe disease (14, 15). Contemporary recommendations also emphasize pulse oximetry as a decision aid, with oxygen saturation (SpO₂) < 90% widely adopted as a critical threshold mandating escalation. Additional red-flag signs recognized in pediatric emergency medicine guidelines include cyanosis, apnea or pauses in breathing, grunting respirations, and inability to speak or breastfeed due to respiratory distress (16, 17). Collectively, these 11 evidence-based red-flag signs form the core set assessed in validated caregiver knowledge instruments and clinical triage protocols across diverse healthcare settings (17, 18). Importantly, although chest-indrawing pneumonia may be managed as an outpatient in tightly defined circumstances, any danger sign or hypoxemia warrants immediate evaluation (14, 19, 20). These clinical anchors translate readily into family-facing education if communicated clearly, for example, stridor at rest is intuitively alarming and, when recognized, can prompt timely care seeking (7, 14, 15). Consequently, population-level preparedness depends not only on facility triage but also on how well caregivers internalize and act upon these danger signals.

China bears a substantial burden of pediatric acute respiratory infections (ARIs) with marked seasonality and evolving post-pandemic pathogen ecology, increasing encounters in which red-flag symptoms may emerge during ostensibly benign illnesses (21, 22). Recent studies describe high virologic positivity among children with ARIs and underscore the predominance of influenza and other respiratory viruses, trends that collectively increase the absolute number of encounters in which red-flag symptoms might emerge during seemingly benign illnesses like acute bronchitis (21, 23, 24). In this context, caregiver awareness of deterioration cues becomes a critical determinant of prehospital timelines and downstream outcomes.

Timely EMS activation in China is mediated by the “120” network, which has undergone digital modernization (e.g., EV-Call 120), although response heterogeneity and geographic inequities persist. These factors shape real-world activation decisions (25–27). These structural realities mean that even when recognition occurs, activation may be constrained by geography, cost perceptions, and systems navigation. Accurate knowledge of the correct number, willingness to call, and confidence that an ambulance will be available, therefore, remain practical determinants of timely prehospital contact, particularly for pediatric respiratory emergencies (27–30).

Evidence from low- and middle-income countries (LMICs) demonstrates recurrent gaps in caregiver recognition of specific respiratory red-flag signs. While fever is commonly identified, cardinal signs—fast breathing, chest indrawing, cyanosis, apnea, and severe lethargy—are frequently under-recognized, and this knowledge gap correlates with inconsistent or delayed care seeking (16, 17). Observational and mixed-methods studies further show that recognition does not always translate into prompt action; delays are shaped by rural residence, low income, lack of insurance, self-medication, perceived illness mildness, and opportunity costs of seeking care (16, 31–33). These findings suggest that educational interventions must be paired with strategies that reduce financial and logistical barriers to calling or presenting for emergency care if meaningful gains in timeliness are to be realized.

Health literacy, a modifiable determinant of caregiver behavior, offers a promising focus for such strategies. The validated three-item Chew screener provides a brief, pragmatic measure correlated with fuller literacy assessments and has been applied across diverse clinical settings (34, 35). Low caregiver health literacy has been associated with non-urgent emergency department utilization and misestimation of illness severity (36, 37). In China, digital health education—often delivered via ubiquitous platforms—has improved parental competencies, underscoring the feasibility of scalable interventions, although respiratory-specific literacy measures linked to time-sensitive actions, such as calling “120” when cyanosis or severe chest indrawing appears, remain under-studied (27, 38–40).

However, against this backdrop, a focused examination of caregivers of children presenting in pediatric settings where acute respiratory illnesses such as bronchitis are commonly encountered is warranted for three reasons. First, acute bronchitis is among the most common pediatric ambulatory diagnoses and serves as a prototypical clinical context in which red-flag features may initially emerge; early recognition in this ostensibly mild context is therefore critical to preventing deterioration (41, 42). Importantly, our study examines caregiver awareness and emergency activation preparedness broadly across caregivers accessing pediatric services, not exclusively those whose children carry a specific bronchitis diagnosis, as the cognitive skills required for danger-sign recognition and timely EMS activation are generalizable across all acute respiratory presentations. Second, most prior work in LMICs has centered on “pneumonia” case definitions or under-five mortality frameworks, with limited disaggregation of red-flag awareness across age-specific vignettes that mirror everyday clinical presentations, and with minimal attention to the cognitive step from recognition to the intention to activate EMS. Third, within China’s maturing yet heterogeneous 120 system, the real-world salience of knowing the ambulance number, and the degree to which costs or transport concerns deter calls, have not been quantified alongside health-literacy gradients and prior respiratory experiences (e.g., a child’s asthma diagnosis or previous hospitalization) in a contemporary single-center urban cohort (20, 27, 43, 44). This study quantifies caregiver awareness of pediatric respiratory red flags and their intended emergency actions in China. We identify key sociodemographic, experiential, and health literacy-related predictors of their knowledge and intended behavior. The impact of perceived barriers and clinical setting on these outcomes is also evaluated. Ultimately, this work seeks to generate actionable evidence for targeted caregiver education and prehospital policies.

## 2 Methods

### 2.1 Study design and setting

This province-wide, cross-sectional study employed a mixed-mode design with dual recruitment channels: (1) facility-based recruitment via QR codes across pediatric departments in Hubei hospitals, and (2) community-based online recruitment through WeChat groups and secure survey platforms. To minimize selection bias, identical questionnaires were administered across modes, with all analyses adjusting for administration method and recruitment channel. Sensitivity analyses compared estimates across collection methods to assess mode effects. Responses were anonymized using sequential study codes with no facility-level identifiers retained. The questionnaire used “study hospital” as a standardized referent for each participant’s usual pediatric healthcare facility, enabling site-neutral data collection while maintaining contextual relevance.

### 2.2 Participants and recruitment

The unit of analysis was the caregiver–child dyad. Eligible volunteers were primary caregivers of children aged 0–12 years residing in Hubei who accessed the survey via onsite QR codes or online links. To align with the study’s focus on general emergency preparedness, eligibility was not contingent upon the child having a specific active respiratory diagnosis at the moment of survey; rather, the study recruited caregivers across pediatric outpatient, emergency, and inpatient settings to capture a representative spectrum of those navigating pediatric respiratory presentations. Inclusion criteria were as follows: (i) primary caregiver (mother, father, grandparent, or other legal guardian) of a child aged 0–12 years; and (ii) completion of the interviewer-administered, assisted self-administered, or online survey during the data collection period. The clinical vignettes and awareness items utilized acute bronchitis and general respiratory distress scenarios as the reference frame for assessing decision-making. Exclusion criteria were as follows: non-primary caregivers (e.g., neighbor and friend), children outside the 0–12-year age range, obvious duplicate submissions (identified by timestamp anomalies and identical response patterns), and inability to complete the survey despite reasonable assistance. A non-identifiable study code linked caregiver and child responses; no direct personal identifiers were recorded.

#### 2.2.1 Sample size and precision rationale

Based on anticipated province-wide accrual via hospital-affiliated outreach and online channels, the study planned enrolment of ≥2,500 caregiver–child dyads to ensure precise estimation of primary proportions. Under the most conservative binomial scenario (*p* = 0.50), *N* = 2,401 yields a 95% confidence-interval half-width of ±2.0% (1.96·√[0.25/N]); the achieved sample (*N* = 2,702) therefore affords ≤ ± 1.9% precision for proportions near 50% and finer precision for more extreme proportions. For multivariable logistic regression, assuming outcome prevalence between 20 and 30%, the sample provides approximately 540–810 events, supporting ≤10 parameters at ≥20 events per variable, consistent with contemporary recommendations for model stability.

### 2.3 Survey instrument and administration

A site-neutral, province-wide questionnaire was used, structured into sections A–I with closed-ended items and 1–5 Likert scales where applicable. Instruments were forward–back translated into Mandarin, piloted with cognitive debriefing, and finalized before fieldwork. Interviewers received standardized training for onsite administration (scripted explanations and laminated vignettes), and the online form mirrored the onsite instrument (identical wording, branching, and validation). Administration mode (face-to-face interviewer vs. assisted self-admin vs. online) and recruitment channel (onsite vs. online) were recorded (C2). The instrument captured site metadata; eligibility; sociodemographic and access characteristics; health literacy (Chew 3-item screener); child respiratory history; awareness of red-flag respiratory signs (E1–E12); intended emergency activation using five age-stratified clinical vignettes (F1–F5); preparedness and prior behavior (G1–G4); and attitudes/perceived barriers (H1–H6). Optional process items (I1–I3) assessed knowledge of local pathways.

### 2.4 Variables and operational definitions

This study prespecified outcomes and covariates and applied uniform operational rules across settings. Throughout, “study hospital” denotes the caregiver’s usual or nearest pediatric hospital used as an anchor in the questionnaire; responses were not tied to specific participating sites.

#### 2.4.1 Primary outcomes

The Awareness Index (AI) was assessed using 12 items (E1–E12). Items E1–E11 comprised 11 true red-flag respiratory signs systematically derived from established international guidelines: the WHO Integrated Management of Childhood Illness (IMCI) framework, which codifies inability to drink, persistent vomiting, convulsions, lethargy/unconsciousness, stridor when calm, and lower chest indrawing as key red-flag signs mandating urgent referral (14, 20); contemporary pediatric respiratory distress assessment criteria emphasizing cyanosis, apnea, grunting, and inability to speak/feed as markers of severe compromise (19, 20); and evidence-based pneumonia management protocols that specify oxygen saturation (SpO₂) < 90% as a critical threshold for immediate escalation (45–47). Additionally, very fast breathing (tachypnea) for age, derived from WHO age-specific respiratory rate thresholds, was included, given its established role as a sensitive pneumonia screening criterion in community and primary care settings (7, 22). Items E1–E11 were true red-flag signs; “Yes” was scored as correct. Item E12 (fever alone without breathing difficulty) functioned as a distractor; “No” was scored as correct. “Do not know” was scored as incorrect. The AI equaled the proportion correct across E1–E12 (range 0–1). The primary adequacy threshold (AI ≥0.70) was specified *a priori* to represent substantial mastery, consistent with conventional educational standards and public-health survey practice; sensitivity thresholds of 0.60 and 0.80 were prespecified. EIAS captured action tendencies across five vignettes (F1–F5). For each vignette, respondents rated the likelihood (1–5) of calling 120, going immediately to the study hospital ED, and waiting/using home remedies; the vignette-specific component was computed as max (call 120, go to ED) minus wait/home (reverse-coded), yielding 1–5. The total EAIS summed components (range 5–25); “High EAIS” was defined *a priori* as ≥20, indicating strong net intention to activate urgent care.

#### 2.4.2 Secondary outcomes

The timing probe after each vignette recorded whether the respondent would act “Immediately,” “≤10 min,” “11–30 min,” or “>30 min”; “Act immediately” was coded as selecting “Immediately.” A composite “appropriate response” was defined for descriptive analysis: for urgent vignettes (F1–F4), Likely/Very likely (4–5) for call 120 and/or ED plus “Act immediately”; for the non-urgent control vignette (F5), Unlikely/Very unlikely (1–2) for call 120 and ED with no immediate action.

#### 2.4.3 Exposures and covariates

Sociodemographic and access characteristics (section B) included caregiver age (years), highest education (None/Primary; Junior Secondary; Senior Secondary; College or above), occupation (homemaker; manual/industrial; service/retail; professional/technical; unemployed; other), monthly household income (site-standard bands), hukou (local vs. non-local), household size (numeric), residence relative to the study hospital anchor (same district; other district in same city; outside city within province; outside province), travel time to the study hospital anchor (minutes), phone/transport availability (Always vs. Sometimes/Never), usual source of pediatric care (study hospital anchor; community health center; other public hospital; private clinic; other), number of visits to the study hospital anchor in the prior 12 months (0; 1–2; 3–4; ≥5), and insurance usable at the study hospital anchor (None; Public; Private/supplemental). Health literacy (section C) used the Chew 3-item screener (sum of C1 and C3 plus reverse-scored C2; range 3–15, with a higher score indicating lower literacy), with categorical bands of High (3–6), Medium (7–9), and Low (10–15) for subgroup analyses. Child respiratory history (section D) included prior respiratory hospitalization at the study hospital anchor (Yes/No), clinician-diagnosed asthma or reactive airway disease (Yes/No/Do not know), and respiratory-related visits in the prior 12 months (0; 1–2; 3–4; ≥5).

Awareness items (section E) comprised the 11 true red-flags—severe chest indrawing/ribs pulling in; cyanosis; apnea/pauses; stridor at rest; grunting; too breathless to speak/cry/drink/breastfeed; unusual sleepiness/lethargy/difficulty waking; convulsions; persistent vomiting precluding fluids; very fast breathing for age; and SpO₂ < 90% if measured—tabulated as correct/incorrect/Do not know. Vignette responses (section F) for call 120, go to ED, and wait/home were dichotomized at 4–5 vs. 1–3 for “Likely/Very likely” analyses; “Act immediately” was derived from the timing probe. Preparedness and prior behavior (section G) yielded a 0–4 Preparedness Score from G1–G4 (High ≥3), and attitudes/perceived barriers (section H) were analyzed as individual items with a barrier count (0–3) derived from endorsements (scores 4–5) of cost (H3), preference to wait (H4), and discouragement by waiting times (H6). Confidence in recognition (H5) was dichotomized as High (4–5) vs. Low (1–3). Knowledge of the EMS number was coded correctly if “120,” and the study setting recorded the recruitment location (OPD, ED, Ward).

### 2.5 Data handling and quality assurance

Survey responses were captured contemporaneously with real-time range checks. Time stamps, completion time, and item-level missingness were logged. The questionnaire required explicit selection for “Do not know” to reduce accidental skips. Quality control procedures for dual-mode data collection included: (i) real-time validation rules programmed into both online platforms (Google Forms and WeChat survey links) and onsite tablet-based instruments, flagging out-of-range values (e.g., child age >144 months and travel time >300 min) and logically inconsistent responses (e.g., college education + age <18 years) for immediate correction; (ii) minimum completion time thresholds, with surveys completed in <3 min flagged for review—manual inspection of response patterns (e.g., straight-lining and implausible vignette responses) led to exclusion of *n* = 47 submissions deemed invalid; (iii) duplicate detection algorithms for online submissions, using IP addresses, device fingerprints, and timestamp clustering to identify potential repeat submissions—*n* = 23 likely duplicates (≥95% identical responses within 24-h window from same IP) were excluded after manual review; (iv) for onsite data collection, trained research assistants underwent standardized protocols including scripted vignette presentation (laminated cards with identical wording), supervised data entry with random 10% double-entry verification (concordance >99.2%), and daily data quality audits by the lead investigator. Missing data were minimal: item-level missingness ranged from 0.3% (caregiver relationship) to 1.8% (monthly household income), with no single variable exceeding 2% missing. The 12 awareness items (E1–E12) and 5 vignettes (F1–F5) had <0.5% missing due to required-response programming. To assess potential mode effects, we compared completion rates, item missingness, and primary outcome prevalence across online versus onsite channels; no significant differences emerged (all *p* > 0.15), suggesting mode did not introduce systematic bias. Because distribution used both onsite and online channels, the dataset retained mode and channel indicators (C2) for analysis; no site identifiers were stored, preventing attribution to specific hospitals. Data were anonymized at collection using the study code.

### 2.6 Statistical analysis

Analyses followed STROBE guidance for cross-sectional studies. Continuous variables were summarized as mean (SD) or median (IQR), and categorical variables as counts and percentages. For awareness items (E1–E12), “Do not know” responses were included in denominators and scored as incorrect. For vignettes (F1–F5), proportions “Likely/Very likely” (4–5) were reported for (i) call 120 and (ii) go to ED; “Act immediately” was derived from the timing probe. The EAIS component per vignette equaled max (call 120, go to ED) minus wait/home (reverse-coded), yielding 1–5; total EAIS summed components (range 5–25). “High EAIS” was defined *a priori* as ≥20. Univariate associations with adequate awareness (AI ≥0.70) and with High EAIS (≥20) were estimated using logistic regression with odds ratios (ORs), 95% confidence intervals (CIs), and two-sided *p*-values. Primary multivariable models were specified *a priori* from substantive literature and a causal framework; robust (Huber–White) standard errors were used.

All multivariable models were adjusted for survey mode and recruitment channel to account for mixed-mode delivery. Logistic regression assumptions were formally assessed as follows: (i) multicollinearity was evaluated using variance inflation factors (VIFs), with VIF < 5 considered acceptable and VIF < 2.5 preferred; all predictors in final models satisfied VIF < 2.5, indicating negligible multicollinearity; (ii) linearity of continuous predictors (caregiver age, child age, travel time, health literacy score) in the log-odds was assessed by comparing models with linear terms versus fractional polynomial transformations—likelihood ratio tests indicated linear specification was adequate (*p* > 0.10 for all comparisons); (iii) influential observations were identified using Cook’s distance and leverage statistics, with sensitivity analyses excluding observations with Cook’s D > 1 yielding materially unchanged estimates. Model fit and discrimination were summarized with the Hosmer–Lemeshow test (*p* > 0.05 indicating acceptable fit) and area under the ROC curve (C-statistic; values >0.70 indicating acceptable discrimination); Nagelkerke pseudo-R^2^ was reported as an additional fit metric.

For the primary awareness model (Table 1), diagnostics were: Hosmer-Lemeshow χ^2^ = 8.42, *p* = 0.394 (good fit); Nagelkerke *R*^2^ = 0.187; AUC = 0.721 (95% CI 0.698–0.744; acceptable discrimination). For the High EAIS model (Table 2), diagnostics were: Hosmer-Lemeshow χ^2^ = 11.3, *p* = 0.185 (good fit); Nagelkerke *R*^2^ = 0.142; AUC = 0.693 (95% CI 0.671–0.715; acceptable discrimination). These metrics confirm that both multivariable models satisfy key assumptions and demonstrate adequate fit and predictive performance.

**Table 1:** Multivariate analysis—adjusted odds ratios for adequate awareness.

Variable	Adjusted OR	95% CI	*p*-value
Education level			
College or above	3.41	2.75–4.23	<0.001
Senior secondary or below	1	–	–
Knowledge of emergency number			
Knows 120	1.67	1.18–2.36	0.004
Incorrect/do not know	1	–	–
Prior respiratory experience			
Prior respiratory hospitalization	1.34	1.06–1.69	0.015
No prior hospitalization	1	–	–
Asthma diagnosis			
Child has an asthma diagnosis	1.28	0.98–1.67	0.069
No asthma diagnosis	1	–	–
Health insurance			
Public insurance	1.26	0.92–1.73	0.154
No/private insurance	1	–	–
Caregiver age			
Per 1-year increase	1.01	0.99–1.02	0.285
Child age			
Per 1-month increase	1	1.00–1.00	0.447
Travel time			
Per 10-min increase	0.98	0.93–1.04	0.512

Model Statistics: Hosmer-Lemeshow goodness-of-fit test: χ^2^ = 8.42, *p* = 0.394 (indicating good model fit); Nagelkerke *R*^2^ = 0.187; Area under ROC curve (C-statistic) = 0.721 (95% CI: 0.698–0.744) (indicating acceptable discrimination). Variance inflation factors for all predictors <2.5, confirming absence of problematic multicollinearity. Linearity of continuous predictors in the log-odds confirmed via fractional polynomial comparisons (all *p* > 0.10).

**Table 2:** Factors associated with high emergency activation intention (EAIS ≥20).

Factor	High EAIS n/N (%)	Low EAIS n/N (%)	Crude OR (95% CI)	Adjusted OR (95% CI)**ᵃ**	*p*-value
Adequate awareness (AI ≥0.70)					
Yes	487/618 (78.8)	131/618 (21.2)	2.67 (2.13–3.35)	2.34 (1.84–2.98)	<0.001
No	1,269/2,084 (60.9)	815/2,084 (39.1)	1.00 (reference)	1.00 (reference)	–
Education level					
College or above	891/1,264 (70.5)	373/1,264 (29.5)	1.52 (1.28–1.81)	1.28 (1.05–1.56)	0.015
Senior secondary or below	865/1,438 (60.2)	573/1,438 (39.8)	1.00 (reference)	1.00 (reference)	–
Preparedness score					
High (≥3)	1,156/1,573 (73.5)	417/1,573 (26.5)	1.89 (1.60–2.24)	1.65 (1.38–1.98)	<0.001
Low (<3)	600/1,129 (53.1)	529/1,129 (46.9)	1.00 (reference)	1.00 (reference)	–
Prior respiratory hospitalization					
Yes	378/519 (72.8)	141/519 (27.2)	1.49 (1.20–1.85)	1.24 (0.98–1.57)	0.073
No	1,378/2,183 (63.1)	805/2,183 (36.9)	1.00 (reference)	1.00 (reference)	–
Confident in recognition					
High confidence (4–5)	1,287/1,854 (69.4)	567/1,854 (30.6)	1.58 (1.33–1.87)	1.42 (1.18–1.71)	<0.001
Low confidence (1–3)	469/848 (55.3)	379/848 (44.7)	1.00 (reference)	1.00 (reference)	–
Study location					
Emergency department	523/740 (70.7)	217/740 (29.3)	1.34 (1.11–1.61)	1.18 (0.96–1.45)	0.114
OPD/ward	1,233/1,962 (62.8)	729/1,962 (37.2)	1.00 (reference)	1.00 (reference)	–

𝐺djusted for all variables shown plus caregiver age, child age, hukou status, insurance type, and health literacy score. Model Statistics: Hosmer-Lemeshow goodness-of-fit test: χ^2^ = 11.3, *p* = 0.185 (indicating good model fit); Nagelkerke *R*^2^ = 0.142; Area under ROC curve (C-statistic) = 0.693 (95% CI: 0.671–0.715) (indicating acceptable discrimination). Variance inflation factors for all predictors <2.5. Linearity assumptions verified for continuous predictors.

#### 2.6.1 Missing data handling

Given minimal item-level missingness (<2% for any variable), complete-case analysis was employed as the primary analytic approach. For multivariable models, observations with missing data on any predictor or outcome were excluded (*n* = 118, 4.4% of enrolled sample), yielding analytic samples of *n* = 2,584 for the awareness model and *n* = 2,589 for the High EAIS model. To assess robustness, we conducted sensitivity analyses using multiple imputations by chained equations (MICE; *m* = 5 imputed datasets, predictive mean matching for continuous variables, logistic regression for binary variables, 50 iterations). Pooled estimates from multiply imputed datasets were materially unchanged from complete-case analyses [e.g., aOR for college education: 3.41 (complete-case) vs. 3.38 (pooled imputation), 95% CI 2.73–4.19], confirming that our findings are robust to missing data assumptions. All analyses reported herein use complete-case data unless otherwise specified.

### 2.7 Reporting

Tables and figures present descriptive statistics, item-level awareness, vignette-specific intentions, and immediate-action proportions, crude and adjusted associations for adequate awareness and High EAIS, subgroup comparisons by setting, and literacy-stratified trends. All analyses used two-sided tests with *α* = 0.05. No adjustments were made for multiplicity in primary analyses; sensitivity analyses at alternative AI thresholds (0.60 and 0.80) contextualize the robustness of education effects on awareness.

## 3 Results

### 3.1 Participant characteristics and study population

A total of 2,702 caregiver–child dyads were enrolled across three clinical settings: 1,542 (57.1%) from the pediatric outpatient department, 740 (27.4%) from the emergency department, and 420 (15.5%) from the pediatric ward. Caregivers were predominantly mothers (62.8%), with a mean age of 35.9 ± 9.6 years (range 18–77 years). Children had a mean age of 58.2 ± 41.6 months (range 1–144 months), with 53.1% male. Nearly half of caregivers (46.8%) had completed college or above education, and 20.9% had junior secondary or less (5.3% none/primary + 15.6% junior secondary). Regarding geographic access, 45.1% of families resided in the same district as the study hospital, with a median travel time of 35 min (IQR 25–53). Most caregivers (88.6%) had public health insurance usable at the study hospital, and 81.6% reported always having access to a phone and transport for emergency situations. The study hospital served as the usual source of pediatric care for 68.2% of participants (Table 3).

**Table 3:** Baseline characteristics and demographics.

Characteristic	n (%) or mean ± SD
Total participants	2,702 (100.0)
Study setting	
Pediatric outpatient department	1,542 (57.1)
Emergency department	740 (27.4)
Pediatric ward	420 (15.5)
Caregiver characteristics	
Age, years (mean ± SD)	35.9 ± 9.6
Age range, years	18–77
Relationship to child	
Mother	1,696 (62.8)
Father	716 (26.5)
Grandparent	242 (9.0)
Other	48 (1.8)
Child characteristics	
Age, months (mean ± SD)	58.2 ± 41.6
Age range, months	1–144
Sex	
Male	1,436 (53.1)
Female	1,266 (46.9)
Geographic access	
Residence relative to the study hospital	
Same district	1,219 (45.1)
Other district (same city)	1,037 (38.4)
Outside the city (same province)	388 (14.4)
Outside province	58 (2.1)
Travel time to hospital, minutes	
Mean ± SD	41.6 ± 23.9
Median (IQR)	35 (25–53)
Socioeconomic characteristics	
Education level	
None/primary	142 (5.3)
Junior secondary	423 (15.6)
Senior secondary	873 (32.3)
College or above	1,264 (46.8)
Monthly household income	
<4 k RMB	421 (15.6)
4–7 k RMB	991 (36.7)
7–12 k RMB	837 (31.0)
>12 k RMB	453 (16.8)
Household registration (hukou)	
Local	1,978 (73.2)
Non-local	724 (26.8)
Occupation	
Professional/technical	751 (27.8)
Service/retail	648 (24.0)
Homemaker	629 (23.3)
Manual/industrial	416 (15.4)
Unemployed	145 (5.4)
Other	113 (4.2)

### 3.2 Healthcare access and respiratory history

Knowledge of China’s emergency ambulance number (120) was high, with 88.3% of caregivers providing the correct response. Health literacy scores averaged 8.4 ± 2.7 on the 3–15 scale (higher scores indicating lower literacy). Prior respiratory hospitalization at the study hospital was reported for 19.2% of children, and 13.4% had a clinician diagnosis of asthma or reactive airway disease. Most families (61.2%) had no respiratory-related visits to the study hospital in the past 12 months (Table 4).

**Table 4:** Healthcare access and health literacy characteristics.

Characteristic	n (%) or mean ± SD
Healthcare access	
Health insurance usable at the study hospital	
Public	2,393 (88.6)
None	162 (6.0)
Private/supplemental	147 (5.4)
Access to phone and transport (always)	2,206 (81.6)
Child’s usual source of pediatric care	
Study hospital	1,843 (68.2)
Community health center	485 (17.9)
Other public hospital	287 (10.6)
Private clinic	87 (3.2)
Number of visits to study hospital (past 12 months)	
0	398 (14.7)
1–2	1,245 (46.1)
3–4	687 (25.4)
≥5	372 (13.8)
Knowledge of emergency services	
Correct ambulance number (120)	2,386 (88.3)
Incorrect ambulance number	316 (11.7)
Health literacy (3-item screener)	
Health literacy score (3–15 scale)ᵃ	
Mean ± SD	8.4 ± 2.7
Range	3–15
Individual items (mean ± SD)	
Need help reading materials (1–5)	2.3 ± 1.2
Confident filling forms (1–5)	3.6 ± 1.1
Hard to understand providers (1–5)	2.5 ± 1.1
Respiratory history	
Prior respiratory hospitalization at study hospital	519 (19.2)
Child diagnosed with asthma/reactive airway disease	363 (13.4)
Respiratory visits to study hospital (past 12 months)	
0	1,654 (61.2)
1–2	756 (28.0)
3–4	198 (7.3)
≥5	94 (3.5)

ᵃHigher scores indicate lower health literacy.

### 3.3 Red-flag respiratory sign awareness

**Figure 1:**
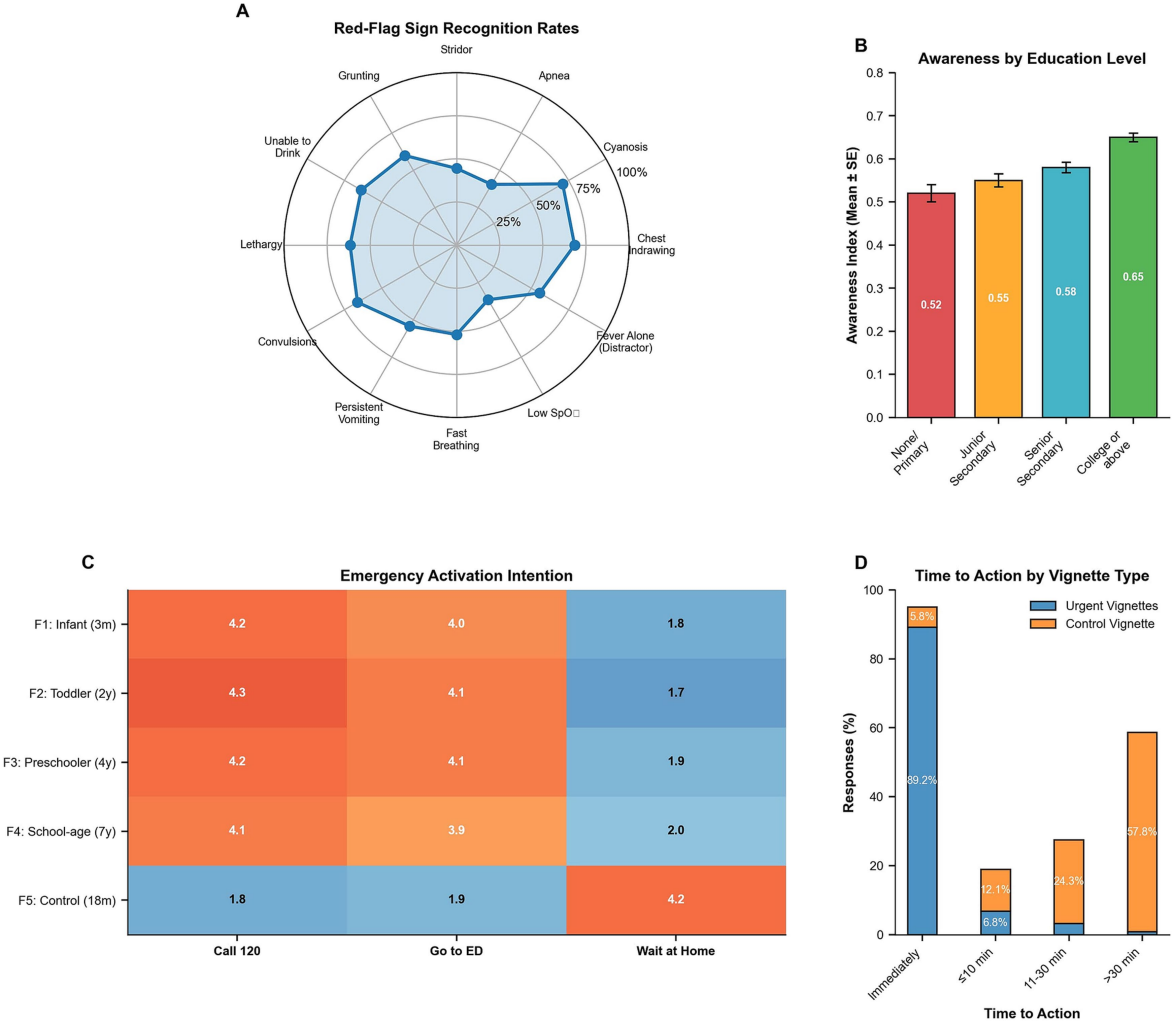
Red-flag respiratory sign recognition and emergency activation patterns: **(A)** recognition rates for individual red-flag signs showing the highest rates for cyanosis and chest indrawing, **(B)** adequate awareness rates by education level, **(C)** emergency activation intention scores across clinical vignettes, and **(D)** immediate action rates for urgent versus non-urgent scenarios.

Recognition rates for the 11 true emergency respiratory signs varied substantially. The highest recognition rates were observed for cyanosis (71.0%) and severe chest indrawing (68.4%). Moderate recognition was seen for convulsions (66.3%), too breathless to speak/drink/breastfeed (63.8%), lethargy (61.7%), and grunting (60.0%). Lower recognition rates were noted for persistent vomiting (54.3%), very fast breathing (51.9%), stridor (44.5%), and apnea (40.7%). The lowest recognition was for very low oxygen saturation (36.6%). For the distractor item (fever alone), 55.6% correctly identified this as not a stand-alone emergency trigger (Table 5; Figure 1A). Overall, 618 out of 2,702 (22.9%) caregivers achieved adequate awareness (AI ≥0.70), while 2,084 out of 2,702 (77.1%) fell below this threshold. By education, adequate awareness was 35.1% among college-or-above caregivers versus 12.1% among ≤senior secondary (Table 6), consistent with the gradient shown in Figure 1B.

**Table 5:** Awareness of red-flag respiratory signs (*N* = 2,702).

Red-flag sign	Correct response n (%)	Do not know n (%)	Incorrect n (%)
True emergency signs			
Severe chest indrawing/ribs pulling in	1,848 (68.4)	523 (19.4)	331 (12.3)
Bluish lips or face (cyanosis)	1,918 (71.0)	398 (14.7)	386 (14.3)
Pauses in breathing/apnea	1,100 (40.7)	892 (33.0)	710 (26.3)
Stridor/harsh noise when breathing at rest	1,202 (44.5)	798 (29.5)	702 (26.0)
Grunting with each breath	1,621 (60.0)	674 (24.9)	407 (15.1)
Too breathless to speak/drink/breastfeed	1,725 (63.8)	612 (22.6)	365 (13.5)
Unusual sleepiness/lethargy	1,666 (61.7)	687 (25.4)	349 (12.9)
Convulsions or seizures	1,791 (66.3)	548 (20.3)	363 (13.4)
Persistent vomiting/cannot keep fluids down	1,468 (54.3)	743 (27.5)	491 (18.2)
Breathing much faster than normal for age	1,401 (51.9)	865 (32.0)	436 (16.1)
Very low oxygen level if measured (SpO₂ < 90%)	990 (36.6)	1,124 (41.6)	588 (21.8)
Distractor item			
Fever alone without breathing difficulty	1,501 (55.6)	568 (21.0)	633 (23.4)

**Table 6:** Factors associated with adequate awareness—univariate analysis.

Factor	Adequate awareness n/N (%)	Inadequate awareness n/N (%)	Crude OR (95% CI)	*p*-value
Education level				
College or above	444/1,264 (35.1)	820/1,264 (64.9)	3.93 (3.23–4.79)	<0.001
Senior secondary or below	174/1,438 (12.1)	1,264/1,438 (87.9)	1.00 (reference)	–
Household registration				
Local hukou	447/1,978 (22.6)	1,531/1,978 (77.4)	0.94 (0.77–1.15)	0.561
Non-local hukou	171/724 (23.6)	553/724 (76.4)	1.00 (reference)	–
Health insurance				
Public insurance	563/2,393 (23.5)	1,830/2,393 (76.5)	1.42 (1.05–1.93)	0.023
No/private insurance	55/309 (17.8)	254/309 (82.2)	1.00 (reference)	–
Phone/transport access				
Always available	496/2,206 (22.5)	1,710/2,206 (77.5)	0.89 (0.71–1.12)	0.325
Sometimes/never	122/496 (24.6)	374/496 (75.4)	1.00 (reference)	–
Knowledge of 120				
Knows the correct number	572/2,386 (24.0)	1,814/2,386 (76.0)	1.85 (1.34–2.57)	<0.001
Incorrect/do not know	46/316 (14.6)	270/316 (85.4)	1.00 (reference)	–
Prior respiratory hospitalization				
Yes	152/519 (29.3)	367/519 (70.7)	1.53 (1.23–1.89)	<0.001
No	466/2,183 (21.3)	1,717/2,183 (78.7)	1.00 (reference)	–
Asthma diagnosis				
Yes	109/363 (30.0)	254/363 (70.0)	1.54 (1.21–1.97)	0.001
No/do not know	509/2,339 (21.8)	1,830/2,339 (78.2)	1.00 (reference)	–
Study location				
Emergency department	179/740 (24.2)	561/740 (75.8)	1.11 (0.91–1.35)	0.317
OPD/ward	439/1,962 (22.4)	1,523/1,962 (77.6)	1.00 (reference)	–
Caregiver type				
Mother	388/1,696 (22.9)	1,308/1,696 (77.1)	1.00 (0.83–1.21)	0.982
Father/other	230/1,006 (22.9)	776/1,006 (77.1)	1.00 (reference)	–

### 3.4 Emergency activation responses to clinical vignettes

Emergency activation intentions were high for the four urgent respiratory scenarios. For the 3-month infant with cough, poor feeding, fast breathing, and chest indrawing (F1), 86.9% indicated they would likely call 120, 79.7% would go to the emergency department, and 89.5% would act immediately. Similar high activation rates were observed for the 2-year-old with cough, blue lips, speech difficulty, and sleepiness (F2: 88.5% call 120, 82.3% ED, 91.8% immediate), the 4-year-old with sudden breathing difficulty and stridor (F3: 86.9% call 120, 82.2% ED, 90.7% immediate), and the 7-year-old with fever, fast breathing, and grunting (F4: 84.6% call 120, 79.5% ED, 88.9% immediate).

In contrast, for the control vignette featuring an 18-month-old with mild cough, runny nose, normal breathing, and playful behavior (F5), appropriate restraint was demonstrated with 12.3% indicating they would call 120, 14.3% would go to the ED, and 5.8% would act immediately. Mean EAIS components were 4.2 (F1), 4.3 (F2), 4.2 (F3), and 4.1 (F4) versus 1.8 (F5) (Table 7; Figure 1C). Pooling “Act immediately” across urgent vignettes F1–F4 using Table 7 yields 90.2% [=(89.5 + 91.8 + 90.7 + 88.9)/4], with 5.8% for the control vignette (Figure 1D).

**Table 7:** Emergency activation intention by clinical vignettes.

Vignette	Call 120 likely/very likely n (%)	Go to ED likely/very likely n (%)	Act immediately n (%)	Mean EAIS component
F1: infant (3 months)				
Cough, poor feeding, fast breathing, chest indrawing	2,348 (86.9)	2,154 (79.7)	2,417 (89.5)	4.2 ± 1.1
F2: toddler (2 years)				
Cough, blue lips, cannot speak, sleepy	2,392 (88.5)	2,223 (82.3)	2,481 (91.8)	4.3 ± 1.0
F3: preschooler (4 years)				
Sudden breathing difficulty, stridor, pauses	2,348 (86.9)	2,220 (82.2)	2,450 (90.7)	4.2 ± 1.1
F4: school-age (7 years)				
High fever, fast breathing, grunting, refuses fluids	2,285 (84.6)	2,147 (79.5)	2,402 (88.9)	4.1 ± 1.2
F5: toddler (18 months)—control				
Cough, runny nose, playful, normal breathing	332 (12.3)	387 (14.3)	156 (5.8)	1.8 ± 1.1

### 3.5 Predictors of adequate awareness

Univariate analysis revealed several significant associations with adequate awareness (AI ≥0.70). College education demonstrated the strongest association (crude OR 3.93, 95% CI 3.23–4.79; *p* < 0.001). Other significant predictors included knowledge of the 120 ambulance number (OR 1.85, 95% CI 1.34–2.57; *p* < 0.001), prior respiratory hospitalization (OR 1.53, 95% CI 1.23–1.89; *p* < 0.001), asthma diagnosis (OR 1.54, 95% CI 1.21–1.97; *p* = 0.001), and public health insurance (OR 1.42, 95% CI 1.05–1.93; *p* = 0.023) (Table 6).

**Figure 2:**
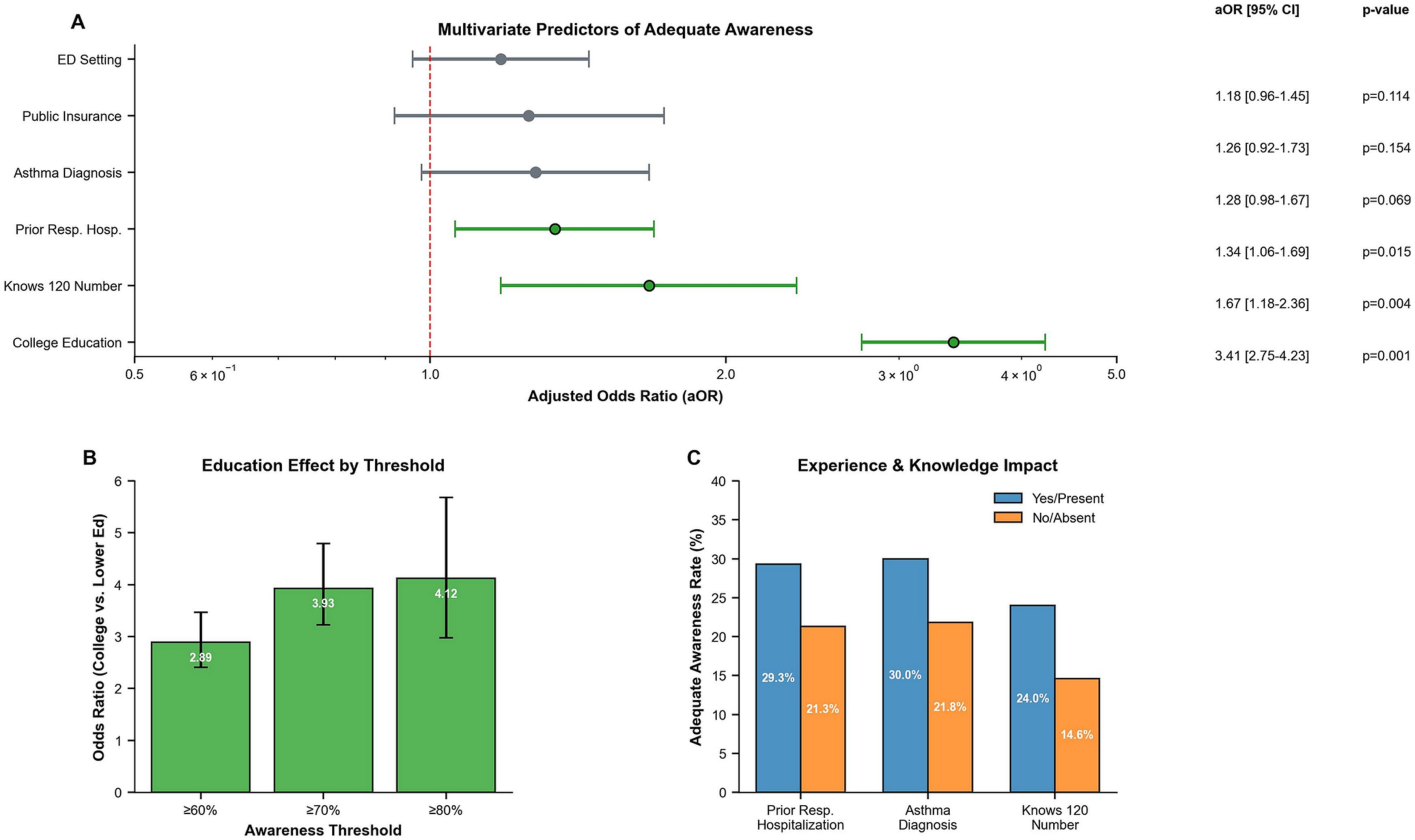
Predictors of adequate awareness: **(A)** forest plot showing adjusted odds ratios for factors associated with adequate awareness (AI ≥0.70), **(B)** education effect consistency across different awareness thresholds, and **(C)** awareness rates by experience and knowledge factors.

In multivariable analysis, college education remained the dominant predictor (aOR 3.41, 95% CI 2.75–4.23; *p* < 0.001). Knowledge of the correct ambulance number (aOR 1.67, 95% CI 1.18–2.36; *p* = 0.004) and prior respiratory hospitalization experience (aOR 1.34, 95% CI 1.06–1.69; *p* = 0.015) also retained independent significance. Asthma diagnosis showed a trending association (aOR 1.28, 95% CI 0.98–1.67; *p* = 0.069). The model demonstrated adequate fit (Hosmer–Lemeshow χ^2^ = 8.42; *p* = 0.394) with moderate discrimination (AUC = 0.721, 95% CI 0.698–0.744) (Table 1; Figure 2A).

The education effect remained robust across different awareness thresholds, with odds ratios of 2.89 for AI ≥0.60, 3.93 for AI ≥0.70, and 4.12 for AI ≥0.80, demonstrating a consistent benefit of higher education regardless of the awareness cut-point selected (Figure 2B). Experience and knowledge factors showed consistent patterns, with caregivers having prior respiratory hospitalization (29.3% vs. 21.3% adequate awareness), asthma diagnosis (30.0% vs. 21.8%), and knowledge of 120 (24.0% vs. 14.6%), demonstrating higher awareness rates (Figure 2C).

### 3.6 Determinants of high emergency activation intention

High EAIS (≥20) was achieved by 1,756 out of 2,702 (65.0%) caregivers. Adequate awareness was strongly associated with high EAIS in both crude (OR 2.67, 95% CI 2.13–3.35) and adjusted analyses (aOR 2.34, 95% CI 1.84–2.98; *p* < 0.001). Additional independent predictors included college education (aOR 1.28, 95% CI 1.05–1.56; *p* = 0.015), high preparedness score (aOR 1.65, 95% CI 1.38–1.98; *p* < 0.001), and high confidence in recognition (aOR 1.42, 95% CI 1.18–1.71; *p* < 0.001) (Table 2).

### 3.7 Subgroup analysis by study setting

**Figure 3:**
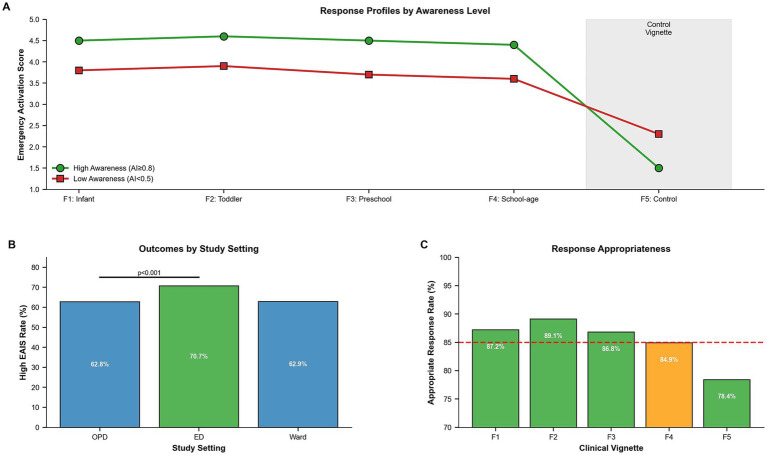
Clinical response patterns: **(A)** emergency activation scores by awareness level, **(B)** setting-specific immediate activation rates, and **(C)** response appropriateness across vignette scenarios.

Significant differences emerged across the three study settings. Emergency department caregivers demonstrated the highest rates of high EAIS (70.7%) compared to outpatient department (62.8%) and ward settings (62.9%) (*p* < 0.001). Adequate awareness rates were similar between outpatient (23.9%) and emergency department (24.2%) settings, with ward caregivers showing lower rates (16.7%) (*p* = 0.003). Emergency department caregivers were more likely to report prior respiratory hospitalization (22.7% vs. 17.2% in OPD; *p* = 0.004) and asthma diagnoses (15.9% vs. 11.5% in OPD; *p* = 0.002). Health literacy scores were lowest (indicating highest literacy) in the outpatient setting (8.2 ± 2.6) compared to the emergency department (8.5 ± 2.7) and ward (8.9 ± 2.9) (*p* < 0.001). Vignette-specific immediate activation rates (“would call 120 immediately”) were consistently highest in the emergency department across all four urgent scenarios (Table 8; Figure 3B).

**Table 8:** Subgroup analysis by study setting.

Characteristic	Pediatric OPD *n* = 1,542 (57.1%)	Emergency department *n* = 740 (27.4%)	Pediatric ward *n* = 420 (15.5%)	*p*-value
Demographics				
Caregiver age, mean ± SD	35.2 ± 9.4	36.8 ± 9.6	37.1 ± 10.2	<0.001
Child age (months), mean ± SD	59.8 ± 42.1	54.2 ± 40.8	58.9 ± 40.9	0.012
College education	782 (50.7)	321 (43.4)	161 (38.3)	<0.001
Local hukou status	1,165 (75.6)	521 (70.4)	292 (69.5)	0.003
Clinical experience				
Prior respiratory hospitalization	265 (17.2)	168 (22.7)	86 (20.5)	0.004
Child has an asthma diagnosis	178 (11.5)	118 (15.9)	67 (16.0)	0.002
Frequent visits (≥5 per year)	198 (12.8)	105 (14.2)	69 (16.4)	0.138
Health literacy and knowledge				
Health literacy score, mean ± SD	8.2 ± 2.6	8.5 ± 2.7	8.9 ± 2.9	<0.001
Knows the correct ambulance number (120)	1,378 (89.4)	648 (87.6)	360 (85.7)	0.056
Primary outcomes				
Adequate awareness (AI ≥0.70)	369 (23.9)	179 (24.2)	70 (16.7)	0.003
High EAIS (≥20)	969 (62.8)	523 (70.7)	264 (62.9)	<0.001
Preparedness and attitudes				
High preparedness score (≥3)	918 (59.5)	433 (58.5)	222 (52.9)	0.043
Confident in recognition (score 4–5)	1,078 (69.9)	501 (67.7)	275 (65.5)	0.149
Cost prevents calling an ambulance	341 (22.1)	178 (24.1)	99 (23.6)	0.502
Emergency activation by setting				
F1: Would call 120 immediately	1,329 (86.2)	665 (89.9)	354 (84.3)	0.004
F2: Would call 120 immediately	1,367 (88.7)	672 (90.8)	353 (84.0)	0.001
F3: Would call 120 immediately	1,341 (87.0)	658 (88.9)	349 (83.1)	0.008
F4: Would call 120 immediately	1,298 (84.2)	636 (86.0)	351 (83.6)	0.363

*p*-values from χ^2^ tests for categorical variables and ANOVA for continuous variables. F1: 3-month infant with cough, poor feeding, very fast breathing with chest indrawing, and noisy breathing at rest. F2: 2-year-old toddler with 2-day cough, blue lips, inability to speak full words, and excessive sleepiness. F3: 4-year preschooler with sudden breathing difficulty, harsh inspiratory sound (stridor) at rest, and brief breathing pauses. F4: 7-year school-age child with high fever, cough, fast breathing with grunting, and refusal to drink.

### 3.8 Health literacy and barriers impact

**Figure 4:**
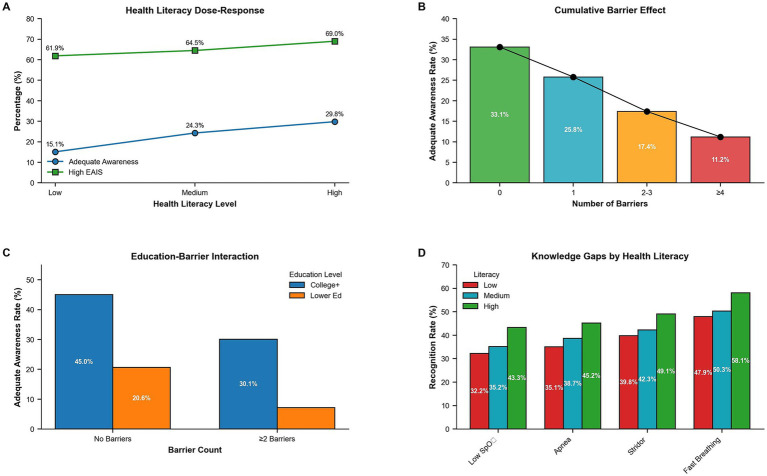
Health literacy impact on outcomes: **(A)** dose–response relationship between health literacy levels and primary outcomes, **(B–D)** item-level recognition rates stratified by literacy level showing progressive decline with lower literacy.

A clear dose–response relationship emerged between health literacy levels and both primary outcomes. Among caregivers with high health literacy (scores 3–6), 29.8% achieved adequate awareness compared to 24.3% with medium literacy (scores 7–9) and 15.1% with low literacy (scores 10–15) (p for trend <0.001). High EAIS rates followed a similar pattern: 69.0% for high literacy, 64.5% for medium literacy, and 61.9% for low literacy (p for trend = 0.031) (Table 9; Figure 4A). In literacy-stratified item performance (Table 9), chest indrawing was correctly recognized by 74.4, 69.0, and 62.3% (high, medium, and low literacy), and cyanosis by 77.0, 71.5, and 65.0%, respectively; low SpO₂ was 43.3, 35.2, and 32.2%, and very fast breathing 58.1, 50.3, and 47.9%. Figures 4B–D further illustrates a monotonic deterioration with accumulating barriers and lower literacy; we report item-level percentages exactly as tabulated in Table 9 and reference Figure 4 for visual trends without introducing additional numeric values not present in the tables.

**Table 9:** Health literacy impact on awareness and emergency activation.

Health literacy level	n (%)	Adequate awareness n (%)	High EAIS n (%)	Mean red-flag score	Key deficits
High literacy (Score 3–6)	823 (30.5)	245 (29.8)	568 (69.0)	7.2 ± 2.1	
Individual awareness rates					
Chest indrawing	–	612 (74.4)	–	–	Lowest deficit
Cyanosis	–	634 (77.0)	–	–	Good recognition
Low SpO₂	–	356 (43.3)	–	–	Major gap
Fast breathing	–	478 (58.1)	–	–	Moderate gap
Medium literacy (score 7–9)	968 (35.8)	235 (24.3)	624 (64.5)	6.8 ± 2.2	
Individual awareness rates					
Chest indrawing	–	668 (69.0)	–	–	Declining recognition
Cyanosis	–	692 (71.5)	–	–	Still good
Low SpO₂	–	341 (35.2)	–	–	Widening gap
Fast breathing	–	487 (50.3)	–	–	Larger deficit
Low literacy (score 10–15)	911 (33.7)	138 (15.1)	564 (61.9)	5.9 ± 2.4	
Individual awareness rates					
Chest indrawing	–	568 (62.3)	–	–	Substantial decline
Cyanosis	–	592 (65.0)	–	–	Notable drop
Low SpO₂	–	293 (32.2)	–	–	Critical gap
Fast breathing	–	436 (47.9)	–	–	Major deficit
Trend analysis					
*p* for linear trend (awareness)	–	<0.001	0.012	<0.001	–
*p* for linear trend (EAIS)	–	<0.001	0.031	<0.001	–
Barrier interaction					
Low literacy + cost barrier	187 (6.9)	18 (9.6)	108 (57.8)	5.1 ± 2.6	Highest risk group
High literacy + no cost barrier	651 (24.1)	211 (32.4)	467 (71.7)	7.5 ± 2.0	Optimal group

Health literacy score: 3–15 scale, where higher scores indicate lower literacy.

### 3.9 Clinical response profiles and appropriateness

Consistent with Figure 3A, caregivers with higher awareness displayed higher emergency activation scores across urgent vignettes and appropriately lower activation for the control vignette, whereas lower-awareness caregivers showed attenuated urgent responses and relatively higher activation for the control scenario. Response appropriateness was high across urgent vignettes with a modestly lower value for the school-age scenario (F4), and lower for the control vignette, indicating some over-activation in non-urgent situations (Figure 3C).

### 3.10 Principal component analysis of caregiver attributes

**Figure 5:**
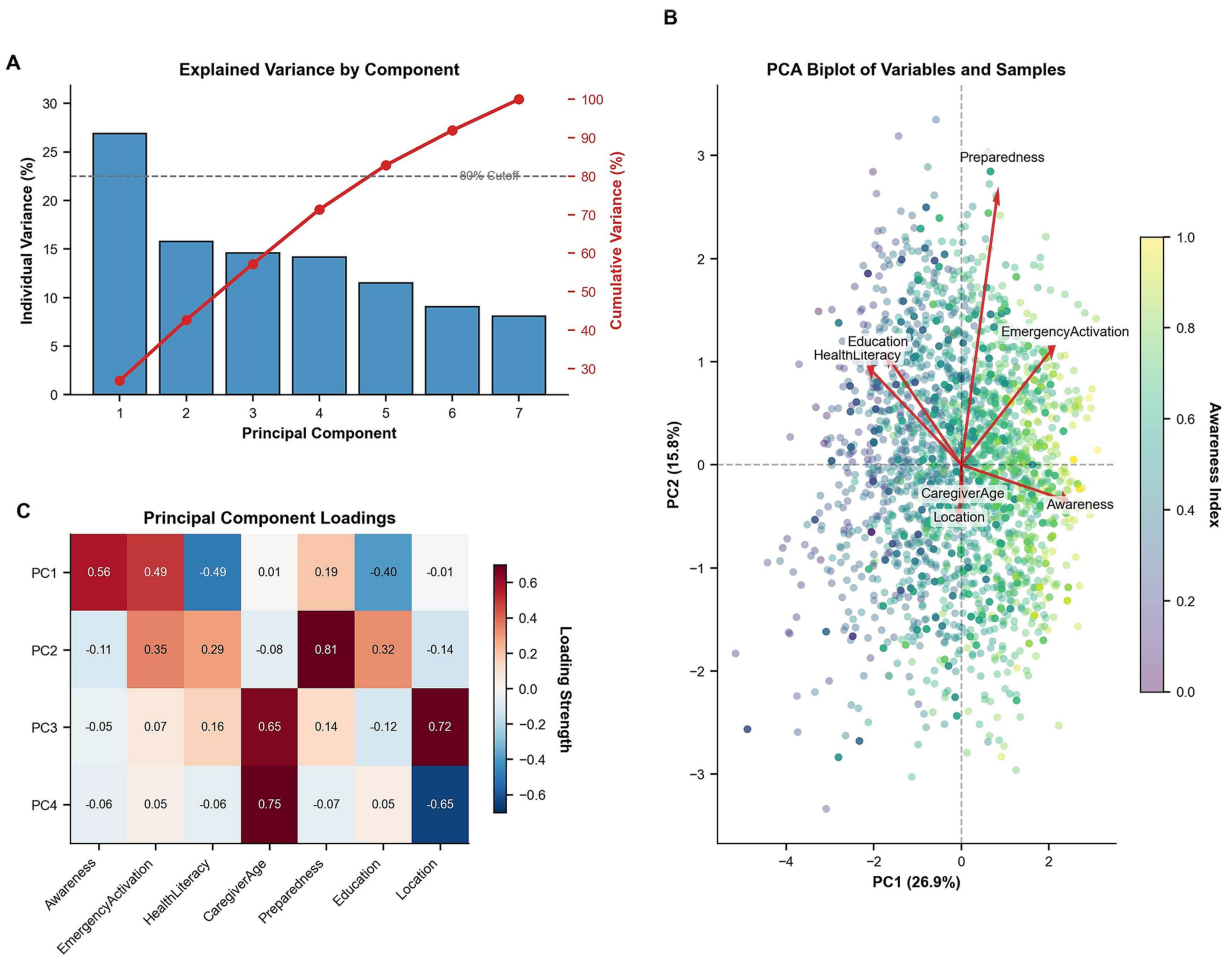
Principal component analysis of caregiver attributes. **(A)** Scree plot showing individual and cumulative variance, with 80% variance explained by component 5. **(B)** Biplot visualizing distinct clustering of high-awareness caregivers (yellow points) aligned with education and preparedness vectors, separated from low-awareness groups (blue points). **(C)** Component loadings identifying latent dimensions: PC1 (knowledge/socioeconomic), PC2 (behavioral readiness), and PC3 (demographic/geographic). These patterns demonstrate the multidimensional nature of preparedness, indicating that behavioral and experiential factors contribute to emergency response capacity independently of socioeconomic status.

To complement regression analyses and explore the multidimensional structure of caregiver preparedness, we performed principal component analysis (PCA) on seven key attributes: awareness index, emergency activation intention, health literacy score, preparedness score, education level, caregiver age, and geographic location. Component loadings were analyzed to identify latent constructs underlying survey responses, and a biplot was generated to visualize the clustering of caregiver phenotypes across these dimensions (Figure 5).

## 4 Discussion

This province-wide, mixed-mode survey of volunteer caregivers in Hubei—distributed via onsite QR codes in pediatric areas and online channels (e.g., WeChat, short links, and a secure Google-Form interface)—delineates a pronounced knowledge–behavior gap in responses to pediatric respiratory emergencies, while simultaneously identifying modifiable correlates that may be leveraged to improve prehospital decision-making. Overall, adequate awareness of red-flag respiratory signs was low (22.9%), with the weakest recognition for apnea, stridor, very low oxygen saturation, and abnormally fast breathing, yet stated emergency activation intentions were appropriately high in life-threatening vignettes and restrained for a non-urgent control. Education, knowledge of the national emergency number, and prior respiratory encounters emerged as salient correlates of awareness and intention, and these gradients persisted across clinical settings. Because responses were anonymous and site-neutral, findings reflect caregiver cognition across Hubei rather than practices of individual hospitals.

The low prevalence of adequate awareness accords with multi-country evidence that caregiver recognition of pneumonia red-flag signs remains suboptimal in low- and middle-income settings, where only about one-third of caregivers identify fast or difficult breathing, and knowledge does not consistently translate into prompt care-seeking after adjustment for confounders (17, 31, 48). In a Brazilian clinic cohort, caregivers recognized common acute respiratory infection symptoms but struggled to appraise severity, mirroring the present study’s steep drop-off for apnea and stridor (16, 48). Similarly, community studies in Africa and Asia report that fever is frequently (mis) perceived as the primary emergency trigger, whereas signs of respiratory failure are under-recognized (18, 31). These convergent observations underscore the salience of symptom salience and prior illness schemas in parental appraisal, wherein visually striking cues (e.g., cyanosis and severe chest indrawing) are more readily detected than physiologically critical yet less familiar phenomena (e.g., apnea or inspiratory stridor).

The particularly low recognition of very low oxygen saturation likely reflects limited household access to pulse oximetry and uncertainty about thresholds, as current guidance and research variably cite referral triggers from SpO₂ < 90% to higher cut-points, and evidence continues to evolve regarding risk at intermediate saturations (45, 46). Programmatic experience shows that introducing oximetry at first contact improves identification of hypoxemia and triage in childhood pneumonia; however, oximetry literacy (what to measure, how to interpret, and when to act) remains largely unaddressed in caregiver education (49, 50). In contrast, the comparatively higher recognition of cyanosis and chest indrawing likely reflects their intuitive visual salience and their longstanding prominence within Integrated Management of Childhood Illness (IMCI) algorithms and pediatric teaching materials (14, 18, 31, 51).

Education demonstrated the strongest association with adequate awareness and remained robust in multivariable models and threshold sensitivity analyses, consistent with prior work linking parental education and health literacy to more accurate symptom appraisal and timelier care-seeking (17, 36, 52). Mechanistically, higher education may enhance general health literacy, numeracy, and navigation self-efficacy, enabling caregivers to integrate multiple cues (effort, rate, color, and behavior) when distinguishing severity. Conversely, lower literacy is consistently associated with non-urgent emergency department utilization, overestimation of illness severity, and preference for immediate evaluation, even when primary care access is available (17, 18, 34, 51–54). The present study’s parallel gradients for awareness and EAIS, therefore, align with a large literature linking literacy-related comprehension to both risk appraisal and health care utilization. Mode-adjusted analyses produced similar estimates, suggesting findings are not artifacts of the survey delivery channel.

The strong associations between awareness and prior respiratory hospitalization or a child’s asthma diagnosis further support an experiential learning pathway. Exposure to severe disease episodes, clinical counselling, and repetition of warning-sign language likely consolidate “illness scripts” that facilitate more accurate recognition of deterioration. Additionally, knowledge of the correct EMS number correlated with both adequate awareness and higher activation intentions, corroborating Chinese EMS research that links the decision to call with public understanding of EMS processes and trust in ambulance services (12, 45, 52). Notably, pre-COVID studies from urban China documented preferences for taxi or private transport over ambulance due to perceived delays, cost, or uncertainty about benefit, while recent digital enhancements such as EV-Call-120 integrate WeChat-based video triage and bidirectional data flow across dispatch, ambulances, and hospitals, which may lower cognitive and logistical barriers to activation (48, 52, 55, 56). Embedding caregiver-facing micro-education (e.g., threshold prompts for breathing rate, indrawing, stridor, and SpO₂) within these platforms could amplify the observed knowledge-to-intention linkage (46, 49).

Vignette responses in the present study suggest that, despite limited aggregate awareness, caregivers nonetheless demonstrate appropriate activation intent in life-threatening scenarios and restraint for non-urgent presentations. This pattern is consonant with qualitative and mixed-methods work, indicating that caregivers often prioritize rapid reassurance and definitive evaluation when confronted with salient respiratory distress, but defer or self-manage milder symptoms; low literacy can shift the threshold toward ED use for ambiguity rather than severity (17, 52, 57, 58). Because recruitment relied on voluntary participation through online and onsite channels, selection may favor caregivers with higher digital access or engagement; nonetheless, the high intention to act in urgent vignettes was consistent across modes. Targeted “teach-to-goal” counselling at ED discharge could therefore convert intention into more calibrated future behavior without exacerbating non-urgent demand.

Gradients by health literacy and perceived barriers revealed monotonic, dose–response relations for both awareness and intention. These gradients align with evidence that literacy constraints hinder the parsing of multi-attribute respiratory severity (rate, work of breathing, mental status, and color) and that financial or transport barriers prolong prehospital delay for pediatric pneumonia (33, 48, 57, 59). Participants reported concerns about direct, uninsured medical costs as a barrier to seeking emergency care (56, 60). Thus, pairing literacy-sensitive messaging with structural enablers—such as dispatch-guided triage, transparent fee schedules, and pre-authorized insurance for pediatric respiratory emergencies—is the most effective strategy for yielding significant gains.

The present data suggest that focusing content on under-recognized but prognostically salient signs—apnea, inspiratory stridor at rest, and hypoxemia—may be especially productive. Principal component analysis confirms the multidimensional architecture of caregiver preparedness. Since PC1 (education and literacy) explains only 26.9% of variance, while behavioral (PC2, 15.8%) and demographic (PC3, 14.1%) dimensions capture substantial independent variance, knowledge-focused interventions alone would leave >70% of preparedness unaddressed. Biplot clustering further identifies high-awareness caregivers as a distinct phenotype with convergent socioeconomic advantages. These findings quantitatively validate our call for multi-component interventions—integrating literacy-sensitive education, experiential simulation, and structural support—to simultaneously address these distinct axes of disadvantage (49, 50).

The clinical and policy implications are threefold, with specific recommendations for targeted health education programs to improve early intervention. First, caregiver education should be reframed from generic respiratory “warning signs” to a short, high-yield triad (apnea/pauses in breathing; stridor at rest or severe indrawing; objective hypoxemia when available), reinforced across venues (OPD, ED, ward) and delivered with simple action thresholds (call 120/go to ED now) (45–47). Educational content should be deliberately tailored to health literacy levels: for low-literacy caregivers, visual aids (pictograms of chest indrawing, video demonstrations of stridor sounds, and color-coded SpO₂ thresholds) paired with teach-back methods have demonstrated efficacy in improving danger-sign recognition and timely care-seeking in low- and middle-income settings (21, 28, 43). For higher-literacy caregivers, structured decision algorithms incorporating multiple clinical cues (respiratory rate for age, work of breathing, mental status, and color) may enhance diagnostic accuracy while preventing over-activation for benign presentations (22, 39). Both approaches should emphasize the three most under-recognized yet prognostically critical signs identified in our study—apnea (40.7% recognition), low oxygen saturation (36.6%), and stridor at rest (44.5%)—given that these features confer particularly high risk of acute deterioration (7, 8, 40, 47). Second, integrating these micro-scripts into EMS e-triage platforms (e.g., EV-Call 120) and discharge instructions can reduce cognitive load at the point of decision (27, 61). Practical implementation strategies include: (i) embedding brief, literacy-appropriate danger-sign checklists into WeChat-based dispatch interfaces that prompt caregivers with yes/no queries (“Is your child’s breathing making a harsh noise even when calm?” “Are you seeing the skin pulling in deeply between the ribs with each breath?”); (ii) automating post-ED-discharge push notifications at 24 and 72 h that reinforce the high-yield triad and provide one-touch “120” activation if deterioration occurs; (iii) piloting QR-code-linked micro-videos (<2 min), demonstrating apnea recognition, stridor sounds, and chest indrawing assessment, distributed during well-child visits and respiratory illness encounters (17, 30–32, 48). These digital strategies align with China’s high mobile penetration and established WeChat health service utilization, offering scalable, low-cost augmentation of traditional face-to-face counseling (31, 32). Third, preparedness elements that were independently associated with EAIS—prior planning, confidence, and equipment readiness—can be operationalized into checklists and brief simulations at routine visits. Specifically, pediatric practices could implement: (i) a one-page “Emergency Readiness Plan” co-created with caregivers at health supervision visits, documenting the correct ambulance number (120), nearest ED location, insurance pre-authorization procedures, and a personalized list of the child’s high-risk features (e.g., history of asthma and prior severe respiratory episode); (ii) 5-min scenario-based simulations during ED discharge or subspecialty visits, wherein clinicians present a brief respiratory deterioration vignette (e.g., “Your child wakes at 2 AM with fast breathing and blue lips—what would you do?”) and provide immediate corrective feedback, a technique shown to improve parental self-efficacy and reduce representation rates (8, 10); (iii) collaboration with community health centers to offer quarterly caregiver workshops combining danger-sign education, hands-on practice with respiratory rate counting and chest indrawing recognition, and facilitated discussion of barriers (cost concerns, transport access, and fear of “bothering” clinicians), thereby addressing both knowledge deficits and structural impediments to timely activation identified in our barrier analysis (25, 37, 44). Given the strong independent associations of prior respiratory hospitalization (aOR 1.34) and asthma diagnosis (aOR 1.28, trending) with adequate awareness in our multivariable models, these high-risk subgroups should be prioritized for intensive, repeated educational interventions at every clinical encounter. Specific, literacy-sensitive education embedded within modernized EMS pathways represents a plausible and testable strategy to compress the interval from symptom recognition to definitive care.

This study has several key strengths. First, we utilized a large, province-wide sample of 2,702 caregiver–child dyads recruited through mixed-mode methodology (onsite and online channels), which enhances the reliability and generalizability of our findings across diverse healthcare settings. Second, the use of validated instruments, including the Chew health literacy screener and standardized clinical vignettes, allowed for precise and objective measurement of both awareness and behavioral intentions. Finally, our study addresses a significant gap in the literature by examining both knowledge and intended emergency activation behaviors in a contemporary Chinese healthcare context, providing new insights into the knowledge–behavior relationship in pediatric respiratory emergencies. Despite its strengths, this study has some limitations that should be acknowledged. The study’s design was cross-sectional and observational, which prevents us from establishing causality between awareness and actual emergency-seeking behaviors. Furthermore, the sample was sourced from voluntary participants in Hubei Province through digital and hospital-based recruitment, which may limit the generalizability of our findings to caregivers without digital access or in other geographic regions. Another potential limitation is reliance on stated intentions rather than observed behaviors during actual emergencies, which could have influenced the results. Future research should aim to address these limitations through prospective studies examining real-world emergency activation behaviors.

## 5 Conclusion

The current study provides compelling evidence that caregiver awareness of pediatric respiratory red-flag signs remains inadequate, particularly for physiologically critical yet less visually apparent signs such as apnea and hypoxemia. Our findings support the hypothesis that educational attainment and health literacy significantly influence both recognition abilities and emergency activation intentions, and highlight the potential importance of targeted, literacy-sensitive education in the context of pediatric emergency preparedness. These results contribute to a deeper understanding of the underlying mechanisms of caregiver decision-making in respiratory emergencies and suggest promising avenues for future clinical interventions integrating brief, high-yield danger sign education with modernized emergency medical service platforms. Further investigation is needed to confirm these findings in prospective studies and explore their translational applications in diverse healthcare systems.

## Data Availability

The raw data supporting the conclusions of this article will be made available by the authors, without undue reservation.
