# Supplementary material for: Caregiver awareness of pediatric respiratory red-flag signs and emergency activation intentions: a province-wide cross-sectional study in China
Source: Front Med (Lausanne). 2026 Mar 12;13:1702865. doi: 10.3389/fmed.2026.1702865 (PMC13018107; doi:10.3389/fmed.2026.1702865)
Supplement: Supplementary file 1 [file Table_1.docx]

## Questionnaire Overview

## Section A: Eligibility and Child Characteristics

A1. Are you the primary caregiver for this child?

- □ Yes (Continue to A2)
  □ No (Ineligible for study)

A2. What is your relationship to the child?

- □ Mother
  □ Father
  □ Grandmother
  □ Grandfather
  □ Other legal guardian (specify): _____________

A3. How old is the child?

- Age: _______ months [If >144 months, ineligible]

A3b. What is the child's sex?

- □ Male
  □ Female

A4. Where does your family currently live (residence relative to study hospital)?

- □ Same district as study hospital
  □ Other district in same city
  □ Outside city but within Hubei Province
  □ Outside Hubei Province

A5. How long does it take to travel from your home to the study hospital?

- _______ minutes

## Section B: Caregiver Sociodemographic Characteristics and Healthcare Access

B1. What is your age?

- _______ years

B2. What is your highest level of education completed?

- □ No formal education or some primary school
  □ Junior secondary (middle school)
  □ Senior secondary (high school)
  □ College or university or above

B3. What is your current occupation?

- □ Homemaker
  □ Manual or industrial worker
  □ Service or retail worker
  □ Professional or technical worker
  □ Unemployed
  □ Other (specify): _____________

B4. What is your household's total monthly income?

- □ Less than 4,000 RMB
  □ 4,000–6,999 RMB
  □ 7,000–11,999 RMB
  □ 12,000 RMB or more

B5. What is your household registration status (hukou)?

- □ Local (registered in this district/city)
  □ Non-local (registered elsewhere)

B6. How many people live in your household?

- _______ people

B7. Where do you usually take your child for pediatric medical care?

- □ Study hospital
  □ Community health center
  □ Other public hospital
  □ Private clinic
  □ Other (specify): _____________

B8. In the past 12 months, how many times have you brought your child to the study hospital for any reason?

- □ 0 times
  □ 1–2 times
  □ 3–4 times
  □ 5 or more times

B9. What type of health insurance does your child have that is usable at the study hospital?

- □ Public insurance (e.g., Urban Employee, Urban-Rural Resident, New Cooperative Medical)
  □ Private or supplemental insurance
  □ No insurance

B10. Do you always have access to a phone and transportation if you needed to seek emergency care for your child?

- □ Always
  □ Sometimes
  □ Never

B11. What is the telephone number to call for an emergency ambulance in China?

- Answer: _____________ [Correct answer: 120]

## Section C: Health Literacy (3-Item Chew Screener)

**Instructions:** Please answer the following questions about reading and understanding health information. For each question, choose the response that best describes your experience.

C1. How often do you need help reading hospital materials or medical instructions?

- □ 1 = Never
  □ 2 = Rarely
  □ 3 = Sometimes
  □ 4 = Often
  □ 5 = Always

C2. How confident are you filling out medical forms by yourself?

- □ 1 = Extremely confident
  □ 2 = Quite confident
  □ 3 = Somewhat confident
  □ 4 = A little confident
  □ 5 = Not at all confident

*Note: This item is reverse-scored (5 becomes 1, 4 becomes 2, 3 stays 3, 2 becomes 4, 1 becomes 5)*

C3. How often do you have difficulty understanding what doctors or nurses tell you about your child's health?

- □ 1 = Never
  □ 2 = Rarely
  □ 3 = Sometimes
  □ 4 = Often
  □ 5 = Always

**Scoring:** Health Literacy Score = C1 + C2(reversed) + C3; Range: 3–15, where higher scores indicate lower health literacy.

**Categories:** High literacy (3–6), Medium literacy (7–9), Low literacy (10–15)

## Section D: Child Respiratory History

D1. Has this child ever been hospitalized at the study hospital for a respiratory problem (such as pneumonia, bronchiolitis, asthma attack, or severe breathing difficulty)?

- □ Yes
  □ No
  □ Don't know

D2. Has a doctor or nurse ever told you that this child has asthma or reactive airway disease?

- □ Yes
  □ No
  □ Don't know

D3. In the past 12 months, how many times have you brought this child to the study hospital for a respiratory-related visit (such as cough, breathing problems, wheezing)?

- □ 0 times
  □ 1–2 times
  □ 3–4 times
  □ 5 or more times

## Section E: Awareness of Red-Flag Respiratory Signs

**Instructions:** We would like to know which signs or symptoms would make you think your child has a breathing emergency that needs immediate medical attention. For each of the following signs in a child with cough or breathing difficulty, please tell us if you think it is an emergency sign.

**Question:** "Which of the following signs in a child with cough or breathing difficulty would make you think it is an emergency that needs immediate medical attention?"

**Response options for each item:** Yes / No / Don't know

1. E1. Severe chest indrawing (ribs pulling in deeply with each breath)

- □ Yes □ No □ Don't know [Correct answer: Yes]

1. E2. Bluish color of the lips or face (cyanosis)

- □ Yes □ No □ Don't know [Correct answer: Yes]

1. E3. Pauses in breathing or stops breathing for several seconds (apnea)

- □ Yes □ No □ Don't know [Correct answer: Yes]

1. E4. Harsh noise when breathing in, even when the child is calm (stridor at rest)

- □ Yes □ No □ Don't know [Correct answer: Yes]

1. E5. Grunting sound with each breath

- □ Yes □ No □ Don't know [Correct answer: Yes]

1. E6. Too breathless to speak, drink, or breastfeed

- □ Yes □ No □ Don't know [Correct answer: Yes]

1. E7. Unusually sleepy, very difficult to wake up, or floppy (lethargy)

- □ Yes □ No □ Don't know [Correct answer: Yes]

1. E8. Convulsions or seizures

- □ Yes □ No □ Don't know [Correct answer: Yes]

1. E9. Persistent vomiting so the child cannot keep fluids down

- □ Yes □ No □ Don't know [Correct answer: Yes]

1. E10. Breathing much faster than normal for the child's age (very fast breathing)

- □ Yes □ No □ Don't know [Correct answer: Yes]

1. E11. Very low oxygen level if measured with a pulse oximeter (oxygen saturation <90%)

- □ Yes □ No □ Don't know [Correct answer: Yes]

1. E12. Fever alone, without any breathing difficulty or other symptoms [DISTRACTOR ITEM]

- □ Yes □ No □ Don't know [Correct answer: No]

**Scoring:** Awareness Index (AI) = Number of correct responses ÷ 12; Range: 0–1. Adequate awareness defined as AI ≥0.70 (≥9 correct responses out of 12).

## Section F: Emergency Activation Intention Vignettes

**Instructions:** We will now describe five different situations involving a child with breathing or cough symptoms. For each situation, please tell us how likely you would be to take different actions and how quickly you would act.

### Vignette 1: Infant (3 months)

**Scenario:** Imagine your 3-month-old baby has had a cough for 2 days. Today, the baby is feeding poorly, breathing very fast, you can see the skin pulling in deeply between the ribs with each breath (severe chest indrawing), and the baby is making noisy breathing sounds even when calm.

F1a. How likely would you be to call the emergency ambulance number 120?

- □ 1 = Very unlikely
  □ 2 = Unlikely
  □ 3 = Neither likely nor unlikely
  □ 4 = Likely
  □ 5 = Very likely

F1b. How likely would you be to take your child immediately to the hospital emergency department?

- □ 1 = Very unlikely
  □ 2 = Unlikely
  □ 3 = Neither likely nor unlikely
  □ 4 = Likely
  □ 5 = Very likely

F1c. How likely would you be to wait at home and use home remedies?

- □ 1 = Very unlikely
  □ 2 = Unlikely
  □ 3 = Neither likely nor unlikely
  □ 4 = Likely
  □ 5 = Very likely

F1d. How quickly would you act?

- □ Immediately (right away)
  □ Within 10 minutes
  □ 11–30 minutes
  □ More than 30 minutes

### Vignette 2: Toddler (2 years)

**Scenario:** Imagine your 2-year-old child has had a cough for 2 days. Today, you notice the child's lips look blue (cyanosis), the child cannot speak full words because of difficulty breathing, and the child seems much more sleepy than usual and is hard to wake up.

F2a. How likely would you be to call the emergency ambulance number 120?

- □ 1 = Very unlikely
  □ 2 = Unlikely
  □ 3 = Neither likely nor unlikely
  □ 4 = Likely
  □ 5 = Very likely

F2b. How likely would you be to take your child immediately to the hospital emergency department?

- □ 1 = Very unlikely
  □ 2 = Unlikely
  □ 3 = Neither likely nor unlikely
  □ 4 = Likely
  □ 5 = Very likely

F2c. How likely would you be to wait at home and use home remedies?

- □ 1 = Very unlikely
  □ 2 = Unlikely
  □ 3 = Neither likely nor unlikely
  □ 4 = Likely
  □ 5 = Very likely

F2d. How quickly would you act?

- □ Immediately (right away)
  □ Within 10 minutes
  □ 11–30 minutes
  □ More than 30 minutes

### Vignette 3: Preschooler (4 years)

**Scenario:** Imagine your 4-year-old child suddenly develops difficulty breathing with a harsh, high-pitched sound when breathing in (stridor at rest), even when calm, and you notice brief pauses where the child stops breathing for a few seconds (apnea).

F3a. How likely would you be to call the emergency ambulance number 120?

- □ 1 = Very unlikely
  □ 2 = Unlikely
  □ 3 = Neither likely nor unlikely
  □ 4 = Likely
  □ 5 = Very likely

F3b. How likely would you be to take your child immediately to the hospital emergency department?

- □ 1 = Very unlikely
  □ 2 = Unlikely
  □ 3 = Neither likely nor unlikely
  □ 4 = Likely
  □ 5 = Very likely

F3c. How likely would you be to wait at home and use home remedies?

- □ 1 = Very unlikely
  □ 2 = Unlikely
  □ 3 = Neither likely nor unlikely
  □ 4 = Likely
  □ 5 = Very likely

F3d. How quickly would you act?

- □ Immediately (right away)
  □ Within 10 minutes
  □ 11–30 minutes
  □ More than 30 minutes

### Vignette 4: School-age (7 years)

**Scenario:** Imagine your 7-year-old child has had a high fever and cough for 2 days. Today, the child is breathing very fast, making a grunting sound with each breath, and refuses to drink any fluids.

F4a. How likely would you be to call the emergency ambulance number 120?

- □ 1 = Very unlikely
  □ 2 = Unlikely
  □ 3 = Neither likely nor unlikely
  □ 4 = Likely
  □ 5 = Very likely

F4b. How likely would you be to take your child immediately to the hospital emergency department?

- □ 1 = Very unlikely
  □ 2 = Unlikely
  □ 3 = Neither likely nor unlikely
  □ 4 = Likely
  □ 5 = Very likely

F4c. How likely would you be to wait at home and use home remedies?

- □ 1 = Very unlikely
  □ 2 = Unlikely
  □ 3 = Neither likely nor unlikely
  □ 4 = Likely
  □ 5 = Very likely

F4d. How quickly would you act?

- □ Immediately (right away)
  □ Within 10 minutes
  □ 11–30 minutes
  □ More than 30 minutes

### Vignette 5: Control Vignette (18 months) [NON-URGENT]

**Scenario:** Imagine your 18-month-old child has had a mild cough and runny nose for 1 day. The child is playful, eating and drinking normally, and breathing at a normal rate without any difficulty.

*Note: This is a control vignette representing a non-urgent situation. Appropriate responses would indicate low likelihood of emergency activation.*

F5a. How likely would you be to call the emergency ambulance number 120?

- □ 1 = Very unlikely
  □ 2 = Unlikely
  □ 3 = Neither likely nor unlikely
  □ 4 = Likely
  □ 5 = Very likely

F5b. How likely would you be to take your child immediately to the hospital emergency department?

- □ 1 = Very unlikely
  □ 2 = Unlikely
  □ 3 = Neither likely nor unlikely
  □ 4 = Likely
  □ 5 = Very likely

F5c. How likely would you be to wait at home and use home remedies?

- □ 1 = Very unlikely
  □ 2 = Unlikely
  □ 3 = Neither likely nor unlikely
  □ 4 = Likely
  □ 5 = Very likely

F5d. How quickly would you act?

- □ Immediately (right away)
  □ Within 10 minutes
  □ 11–30 minutes
  □ More than 30 minutes

**Scoring for Section F:**

Emergency Activation Intention Score (EAIS) Component per vignette = max(Call 120 score, Go to ED score) − Wait/Home score (reverse-coded); Range per vignette: 1–5

Total EAIS = Sum of components across all 5 vignettes; Range: 5–25

High EAIS defined as ≥20 (indicating strong net intention to activate urgent care)

"Act immediately" = Selecting "Immediately" for time-to-action question

## Section G: Preparedness and Prior Behaviors

G1. Have you made a plan for where you would take your child if they had a breathing emergency (e.g., knowing which hospital emergency department to go to)?

- □ Yes
  □ No

G2. Have you ever called the emergency ambulance service (120) to transport your child to the study hospital?

- □ Yes
  □ No

G3. Do you keep a thermometer or rescue medications (such as fever medicine or breathing treatments) at home for your child?

- □ Yes
  □ No

G4. Has a doctor or nurse at the study hospital ever counseled you about warning signs of serious breathing problems in children?

- □ Yes
  □ No

**Scoring:** Preparedness Score = Sum of "Yes" responses to G1–G4; Range: 0–4. High preparedness defined as score ≥3.

## Section H: Attitudes and Perceived Barriers to Emergency Care Seeking

**Instructions:** For each of the following statements, please indicate how much you agree or disagree using a scale from 1 to 5, where 1 = Strongly disagree and 5 = Strongly agree.

H1. Calling an ambulance is the best way to get a very sick child to the hospital quickly.

- □ 1 = Strongly disagree
  □ 2 = Disagree
  □ 3 = Neither agree nor disagree
  □ 4 = Agree
  □ 5 = Strongly agree

H2. In our city, ambulances are reliable and arrive quickly.

- □ 1 = Strongly disagree
  □ 2 = Disagree
  □ 3 = Neither agree nor disagree
  □ 4 = Agree
  □ 5 = Strongly agree

H3. The cost of calling an ambulance would prevent me from using it, even in an emergency.

- □ 1 = Strongly disagree
  □ 2 = Disagree
  □ 3 = Neither agree nor disagree
  □ 4 = Agree
  □ 5 = Strongly agree

H4. I would prefer to wait and see if my child gets better before going to the emergency department.

- □ 1 = Strongly disagree
  □ 2 = Disagree
  □ 3 = Neither agree nor disagree
  □ 4 = Agree
  □ 5 = Strongly agree

H5. I feel confident that I can recognize when my child's breathing problem is serious enough to need emergency care.

- □ 1 = Strongly disagree
  □ 2 = Disagree
  □ 3 = Neither agree nor disagree
  □ 4 = Agree
  □ 5 = Strongly agree

H6. Long waiting times at the emergency department discourage me from taking my child there.

- □ 1 = Strongly disagree
  □ 2 = Disagree
  □ 3 = Neither agree nor disagree
  □ 4 = Agree
  □ 5 = Strongly agree

**Barrier Count:** Number of barriers endorsed (scoring 4–5 on items H3, H4, and H6); Range: 0–3

**Confidence in Recognition:** High confidence defined as H5 score of 4–5; Low confidence as H5 score of 1–3

## Section I: Optional Process and Pathway Knowledge Items

*Note: These items were optional and used to assess knowledge of local emergency care pathways. Not all participants received these questions.*

I1. Is the study hospital emergency department open 24 hours a day, 7 days a week?

- □ Yes
  □ No
  □ Don't know

I2. If you brought your child to the study hospital emergency department with a breathing emergency, do you think they would be seen immediately by a doctor?

- □ Yes
  □ No
  □ Don't know

I3. Does the study hospital have a separate pediatric area or pediatric specialists available in the emergency department?

- □ Yes
  □ No
  □ Don't know

## End of Questionnaire

Thank you for completing this survey. Your responses will help us understand how caregivers recognize and respond to breathing emergencies in children, and will inform the development of educational programs to improve pediatric emergency care.

## Administration Notes

- Survey completion time: Median 12 minutes (IQR 9–16 minutes)
- Mode of administration: Face-to-face interviewer-administered, assisted self-administered, or online
- Language: Mandarin Chinese (forward-back translation from English)
- Pilot testing: Cognitive debriefing with n=15 caregivers prior to fieldwork
- Onsite administration: Laminated vignette cards with standardized scripted presentation
- Online administration: Identical wording and branching logic to onsite instrument
- Quality control: Real-time validation, minimum completion time thresholds, duplicate detection algorithms
